# A Novel Histone Deacetylase Inhibitor Exhibits Antitumor Activity via Apoptosis Induction, F-Actin Disruption and Gene Acetylation in Lung Cancer

**DOI:** 10.1371/journal.pone.0012417

**Published:** 2010-09-14

**Authors:** Yen-An Tang, Wei-Ling Wen, Jer-Wei Chang, Tzi-Tang Wei, Yi-Hung Carol Tan, Santosh Salunke, Chien-Tien Chen, Ching-Shih Chen, Yi-Ching Wang

**Affiliations:** 1 Institute of Basic Medical Science, National Cheng Kung University, Tainan, Taiwan, Republic of China; 2 Department of Life Science, National Taiwan Normal University, Taipei, Taiwan, Republic of China; 3 Department of Pharmacology, National Cheng Kung University, Tainan, Taiwan, Republic of China; 4 Department of Chemistry, National Taiwan Normal University, Taipei, Taiwan, Republic of China; 5 Department of Chemistry, National Tsing Hua University, Hsinchu, Taiwan, Republic of China; 6 Division of Medicinal Chemistry and Pharmacognosy, College of Pharmacy, The Ohio State University, Columbus, Ohio, United States of America; University of Hong Kong, Hong Kong

## Abstract

**Background:**

Lung cancer is the leading cause of cancer mortality worldwide, yet the therapeutic strategy for advanced non-small cell lung cancer (NSCLC) is limitedly effective. In addition, validated histone deacetylase (HDAC) inhibitors for the treatment of solid tumors remain to be developed. Here, we propose a novel HDAC inhibitor, OSU-HDAC-44, as a chemotherapeutic drug for NSCLC.

**Methodology/Principal Findings:**

The cytotoxicity effect of OSU-HDAC-44 was examined in three human NSCLC cell lines including A549 (p53 wild-type), H1299 (p53 null), and CL1-1 (p53 mutant). The antiproliferatative mechanisms of OSU-HDAC-44 were investigated by flow cytometric cell cycle analysis, apoptosis assays and genome-wide chromatin-immunoprecipitation-on-chip (ChIP-on-chip) analysis. Mice with established A549 tumor xenograft were treated with OSU-HDAC-44 or vehicle control and were used to evaluate effects on tumor growth, cytokinesis inhibition and apoptosis. OSU-HDAC-44 was a pan-HDAC inhibitor and exhibits 3–4 times more effectiveness than suberoylanilide hydroxamic acid (SAHA) in suppressing cell viability in various NSCLC cell lines. Upon OSU-HDAC-44 treatment, cytokinesis was inhibited and subsequently led to mitochondria-mediated apoptosis. The cytokinesis inhibition resulted from OSU-HDAC-44-mediated degradation of mitosis and cytokinesis regulators Auroroa B and survivin. The deregulation of F-actin dynamics induced by OSU-HDAC-44 was associated with reduction in RhoA activity resulting from srGAP1 induction. ChIP-on-chip analysis revealed that OSU-HDAC-44 induced chromatin loosening and facilitated transcription of genes involved in crucial signaling pathways such as apoptosis, axon guidance and protein ubiquitination. Finally, OSU-HDAC-44 efficiently inhibited A549 xenograft tumor growth and induced acetylation of histone and non-histone proteins and apoptosis *in vivo*.

**Conclusions/Significance:**

OSU-HDAC-44 significantly suppresses tumor growth via induction of cytokinesis defect and intrinsic apoptosis in preclinical models of NSCLC. Our data provide compelling evidence that OSU-HDAC-44 is a potent HDAC targeted inhibitor and can be tested for NSCLC chemotherapy.

## Introduction

Lung cancer is the leading cause of cancer mortality worldwide. The 5-year overall survival of non-small cell lung cancer (NSCLC) is less than 15% in many countries [1], [2]. The standard therapeutic strategy for advanced NSCLC is platinum-based double-agent chemotherapy which, however, has reached a plateau of potency in improving survival of patients [3], [4]. Only a few “target agents” have showed benefits when used in combination with platinum-based double-agent for NSCLC chemotherapy, such as bevacizumab, erlotinib and gefitinib, in a subset of patients [5], [6]. Therefore, the development of novel molecular targeted drugs with more general effectiveness for lung cancer patients is an imperative task.

The epigenetic changes as well as genetic alterations are associated with tumorigenesis [7]. A recent report identifies that the epigenetic changes involving modifications of histones H2A and H3 in NSCLC patients influence the overall survival and disease-free survival, providing the prognostic value of histone modifications [8]. It also reveals the rationale for the use of drugs against histone modification as a therapeutic strategy for NSCLC.

Histone deacetylases (HDACs) are the enzymes that catalyze the deacetylation of histones and epigenetically regulate chromatin architecture and gene expression. It has been demonstrated that inhibition of HDACs reverses aberrant epigenetic status and exhibits potent antitumor activities by inducing cell cycle arrest, differentiation and/or apoptosis in diverse cancer cells [9], [10]. HDAC inhibitors are classified into six groups according to their chemical structures and at least 12 of them have progressed to clinical trials [9], [11]. To date, the U.S. Food and Drug Administration approves two HDAC inhibitors, vorinostat (SAHA, suberoylanilide hydroxamic acid, Zolinza®) and romidepsin (FK228, depsipeptide, Istodax®), for the treatment of cutaneous manifestations of cutaneous T-cell lymphoma (CTCL) [12]. However, some adverse events occur in patients treated with vorinostat or other HDAC inhibitors, which may result from the high concentrations of dose used during the treatment for solid tumors in clinical trials [11], [13].

In the present study, we propose a novel class of potent phenylbutyrate-based HDAC inhibitor, OSU-HDAC-44 [4-(2,2-dimethyl-4-phenyl-butyrylamino)-*N*- hydroxy-benzamide], a derivative of known HDAC inhibitor, *N*-Hydroxy-4-(4-phenylbutyryl-amino)benzamide (HTPB) [14]. The antitumor activities and mechanisms of OSU-HDAC-44 were studied in NSCLC cell and mice xenograft models. We found that OSU-HDAC-44 was a pan-HDAC inhibitor and exhibited 3-4 times more effectiveness in suppressing cell proliferation *in vitro* and tumor growth *in vivo* compared to SAHA or trichostatin A (TSA). In addition, OSU-HDAC-44 induced mitosis and cytokinesis defect followed by mitochondria-mediated apoptosis in both cell and animal models. Chromatin-immunoprecipitation-on-chip analysis revealed the genome-wide target genes which were induced by OSU-HDAC-44-mediated hyperacetylation of chromatin. Our data suggest that OSU-HDAC-44 was an HDAC inhibitor and could be applied as targeted anticancer drug for NSCLC chemotherapy.

## Results

### OSU-HDAC-44 inhibits cell proliferation and shows synergistic effects with cisplatin regardless of p53 status

**Figure 1:**
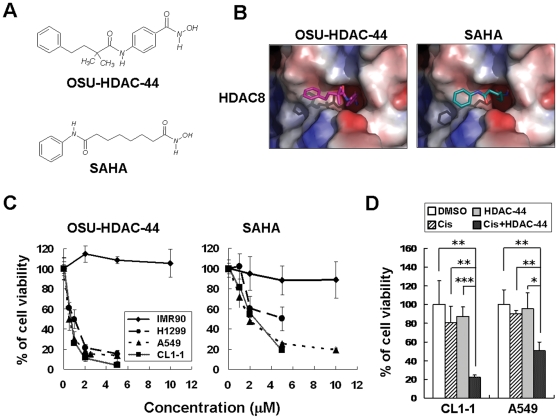
Chemical structure, molecular docking analysis, and the effect of OSU-HDAC-44 on cell viability. (**A**) Chemical structure of OSU-HDAC-44 and SAHA. (**B**) Molecular docking analysis of OSU-HDAC-44 and SAHA. The structures of OSU-HDAC-44 and SAHA were calculated and the docking mode on catalytic domain of HDAC8 was predicted using the docking program GOLD 4.0.1. (**C**) Dose-dependent effects of OSU-HDAC-44 (*left*) and SAHA (*right*) on cell viability in IMR90, H1299, A549 and CL1-1 cells. Cells were treated with 0.5–10 µM of OSU-HDAC-44 or SAHA for 48 h, and cell viability was assessed by trypan blue exclusion assay. (**D**) OSU-HDAC-44 synergized with cisplatin to suppress cell proliferation. Cells were exposed to cisplatin (Cis) alone for 4 h, OSU-HDAC-44 (HDAC-44) alone for 48 h, or pretreated with OSU-HDAC-44 for 48 h before cisplatin treatment for 4 h, and then drug were withdrew and cells were incubated with drug-free media for additional 48 h. Cell viability was assessed by trypan blue exclusion assay. CL1-1 cells were treated with 4.4 µM cisplatin or 0.3 µM OSU-HDAC-44. A549 cells were treated with 1.6 µM cisplatin or 0.2 µM OSU-HDAC-44. Data represent mean ± SEM from three independent experiments. * *P*<0.05; ** *P*<0.01; *** *P*<0.001.

The structure of OSU-HDAC-44 and SAHA are shown in Fig. 1A. Docking analysis demonstrated that OSU-HDAC-44 interacted with the catalytic domain of HDAC 8, suggesting the direct function of OSU-HDAC-44 in targeting HDACs (Fig. 1B).

The cell growth inhibition activities of OSU-HDAC-44 were assessed in three human NSCLC cell lines including A549 (p53 wild-type), H1299 (p53 null), and CL1-1 (p53 mutant). SAHA was included as a positive control HDAC inhibitor. OSU-HDAC-44 significantly inhibited cell proliferation in all cancer cell lines despite their differences in p53 background, and did not cause apparent cytotoxicity to IMR90 cells, a normal lung cell line (Fig. 1C). OSU-HDAC-44 suppressed cell viability of A549 and CL1-1 cells with submicromolar IC_50_ values (0.65±0.08 and 0.67±0.01 µM, respectively) and the IC_50_ value of H1299 were extrapolated to be 1.14±0.14 μM. Notably, OSU-HDAC-44 exhibited 3–4 times more potency than SAHA in anticancer capacity (IC_50_: A549, 1.90±0.16; CL1-1, 2.85±0.27; H1299, 4.87±0.98 µM). In addition, Fig. 1D showed that OSU-HDAC-44 acted in synergy with cisplatin to enhance cell death in CL1-1 and A549 cells, which were both cisplatin-resistant cells.

### OSU-HDAC-44 induces cytokinesis inhibition and apoptosis

**Figure 2:**
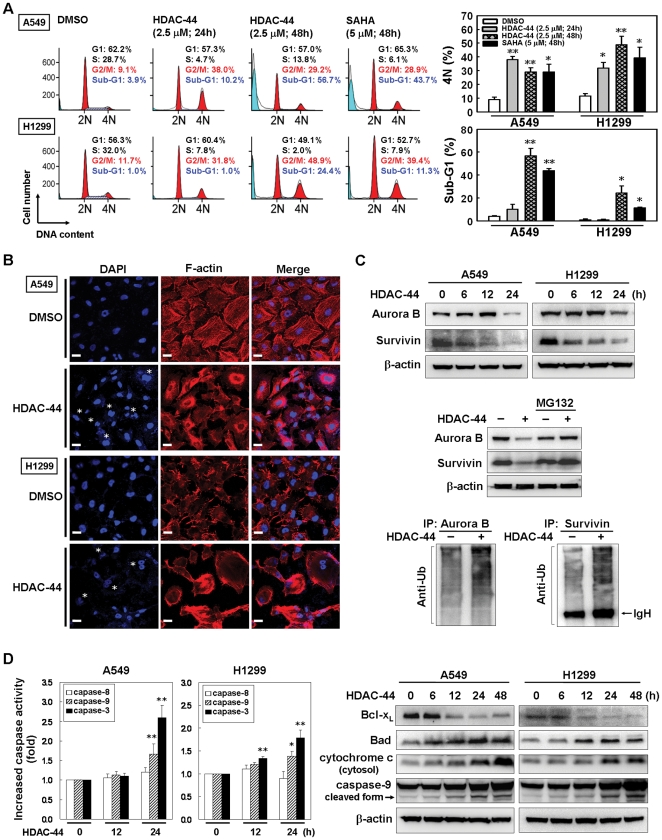
OSU-HDAC-44 induces cytokinesis inhibition and subsequently leads to intrinsic apoptosis. (**A**) The effects of OSU-HDAC-44 on cell cycle distribution in A549 and H1299 cells. Cells were treated with 2.5 µM OSU-HDAC-44 or 5 µM SAHA for indicated times and assessed by flow cytometry. *Left*, results from one representative experiment are shown. *Right*, the mean percentage of G2/M and sub-G1 fraction population is plotted in the histogram. (**B**) The bi-nucleated cells and dysregulation of F-actin induced by OSU-HSAC-44. Cells were treated with 2.5 µM OSU-HDAC-44 for 48 h, and then fixed and stained with DAPI (DNA) and phalloidin (F-actin). **Asterisk** pointed to the bi-nucleus. Scale bars: 30 µm. (**C**) OSU-HDAC-44 induced degradation of Aurora B and survivin via 26S proteasome pathway. *Upper*, time-dependent decreases in Aurora B and survivin protein levels after 2.5 µM OSU-HDAC-44 treatment. *Middle*, A549 cells were treated with 2.5 µM OSU-HDAC-44 in the presence or absence of MG132 for 24 h. *Lower*, A549 cells were treated with 2.5 µM OSU-HDAC-44 for 24 h and cell lysate was subjected to IP assay using anti-Aurora B or anti-survivin specific antibodies and blotted with anti-ubiquitination antibody (Anti-Ub). (**D**) Caspase activity assay (*left)* and Western blot analyses (*Right)* confirmed that OSU-HDAC-44 induced intrinsic apoptosis pathway. Cells were treated with 2.5 µM OSU-HDAC-44 for indicated times and the subjected to caspase activity assay and Western blot analyses. Data represent mean ± SEM from three independent experiments. * *P*<0.05; ** *P*<0.01.

To investigate the underlying mechanism of cell growth repression by OSU-HDAC-44, the effects of OSU-HDAC-44 on cell cycle progression were assessed by flow cytometry. Treatment with 2.5 µM OSU-HDAC-44 for 24 hours caused A549 and H1299 cells to accumulate in G2/M phase (4N cells), and subsequently led to apoptosis (sub-G1 cells) at 48 hours treatment, while exposure to higher concentration (5 µM) of SAHA for 48 hours had similar effect (Fig. 2A), indicating that OSU-HDAC-44 exerted a more potent cell cycle deregulation effect than did SAHA. To examine the cellular consequences of OSU-HDAC-44-mediated accumulation of 4N cells, time-lapse microscopic analyses were performed. As shown in Fig. S1A, OSU-HDAC-44 caused the appearance of the defective cleavage furrow structure and the two daughter cells were fused back together, while untreated cells passed normally through cell division. Concordantly, about 20% cells treated with OSU-HDAC-44 were accumulated as bi-nucleated cells, compared with less than 5% of control cells (Fig. 2B and Fig. S1B). OSU-HDAC-44 also caused micronuclei formation and disrupted the normal structure of F-actin of A549 and H1299 cells (Fig. 2B). Hence, these results suggested that OSU-HDAC-44 may cause aberrant cytokinesis and subsequently led to apoptosis in lung cancer cells.

To identify the molecular mechanism involved in OSU-HDAC-44 induced cytokinesis inhibition, the cell cycle-regulatory proteins were examined. The oscillation of mitotic inhibitor Weel and mitotic markers phosphorylated histone H3 and cyclin B expression indicated that OSU-HDAC-44-treated cells were in M phase after 12 hours treatment and subsequently exited M phase (Fig. S1C), accompanied with cytokinesis defect. Moreover, OSU-HDAC-44 caused decreases in protein levels of Aurora B and survivin (Fig. 2C; upper), which are essential for the progression of mitosis and cytokinesis [15], [16]. Notably, OSU-HDAC-44 induced ubiquitination of Aurora B and survivin, and cotreatment with proteosome inhibitor MG132 prevented the OSU-HDAC-44-induced degradation of Aurora B and survivin (Fig. 2C; middle and lower). Next, we used nocodazole to synchronize cells at pre-metaphase and to further confirm that OSU-HDAC-44 indeed triggered abnormal degradation of Aurora B and survivin at mitotic phase. As shown in Fig. S1D and E, treatment with nocodazole for 24 hours caused accumulation in Aurora B and survivin proteins, whereas combination of OSU-HDAC-44 and nocodazole resulted in decreases Aurora B and survivin protein levels upon 24 hours post-treatment. These results suggested that the OSU-HDAC-44-mediated failure of cytokinesis may partly result from the downregulation of Aurora B and survivin proteins via 26S proteasome pathway.

### OSU-HDAC-44 activates the intrinsic apoptotic pathway

To further elucidate the OSU-HDAC-44-induced apoptosis, we performed phosphatidylserine (PS) staining analyses to detect the early process of apoptosis. As shown in Fig. S2, OSU-HDAC-44 treatment for 24 hours increased the intensity of PS staining in contrast to low staining intensity upon DMSO treatment in A549 and H1299 cells. In addition, OSU-HDAC-44 treatment significantly stimulated caspase-3 and caspase-9 (an indicator of the intrinsic mitochondrial pathway) activities after 24 hours treatment whereas the activity of caspase-8 (an indicator of the extrinsic membrane receptor pathway) remained unaffected in A549 and H1299 cells (Fig. 2D, left). Moreover, treatment with 2.5 µM OSU-HDAC-44 for 12 hours caused a decrease in anti-apoptotic protein Bcl-x_L_, while it increased the pro-apoptotic protein, Bad, within 6–12 hours treatment in A549 and H1299 cells (Fig. 2D, right). The release of cytochrome c into the cytosol accompanied by the cleavage of pro-caspase-9 was also seen after OSU-HDAC-44 treatment for 24–48 hours. These results further confirmed that OSU-HDAC-44 could induce the intrinsic apoptotic pathway in lung cancer cells.

### OSU-HDAC-44 induces protein acetylation with its ability to target numerous HDACs

**Figure 3:**
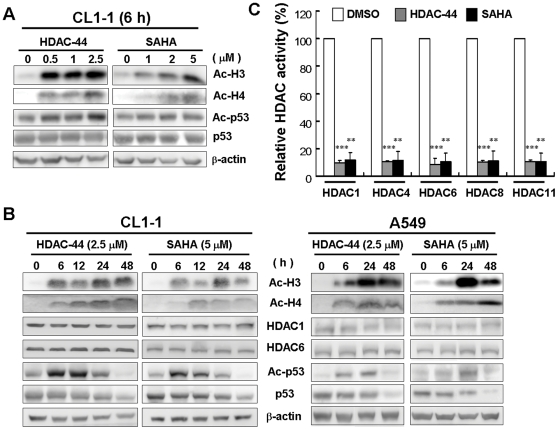
Effect of OSU-HDAC-44 on the biomarkers associated with broad inhibition on numerous HDACs. Dose-dependent effects (**A**) and time-dependent effects (**B**) of OSU-HDAC-44 on the histone and non-histone proteins. Ac-H3, acetylated histone H3; Ac-H4, acetylated histone H4; Ac-p53, acetylated p53; p53, total p53. (**C**) OSU-HDAC-44 suppressed activities of class I (HDAC1 and HDAC8), class II (HDAC4 and HDAC6), and class IV (HDAC11) HDACs. Different HDAC isotypes were immunoprecipitated from H1299 nuclear extract by specific antibodies, and then subjected to *in vitro* HDAC inhibition assay as described in Materials and Methods section. Data represent mean ± SEM from three independent experiments. ** *P*<0.01; *** *P*<0.001.

The biomarkers of HDAC inhibition are acetylation of histone and non-histone proteins, and induction p21^Cip1^ expression in a p53-independent manner [17], [18]. Exposure to OSU-HDAC-44 induced acetylation of histone H3, histone H4 and p53 in a dose-dependent manner (Fig. 3A) and time-dependent manner (Fig. 3B), while it did not affect the HDAC1 and HDAC6 protein levels (Fig. 3B and Fig. S3). Notably, such effects were greater compared to that of SAHA. Despite the p53 status, OSU-HDAC-44 induced the expression of p21^Cip1^ mRNA and protein in A549 and H1299 cells (Fig. S4). To examine the target specificity of OSU-HDAC-44 on class I, II, and IV HDACs, *in vitro* HDAC inhibition assay was performed. As shown in Fig. 3C, the deacetylase activities of different HDAC isotypes including class I (HDAC1 and HDAC8), class II (HDAC4 and HDAC6), and class IV (HDAC11) were significantly inhibited by OSU-HDAC-44. Such effects were greater compared to that of SAHA, a known pan-HDAC inhibitor. These results suggested that OSU-HDAC-44 induced protein acetylation by exerting broad inhibitory activity upon numerous HDACs.

### OSU-HDAC-44 increases gene expression by loosening the chromatin structure

**Figure 4:**
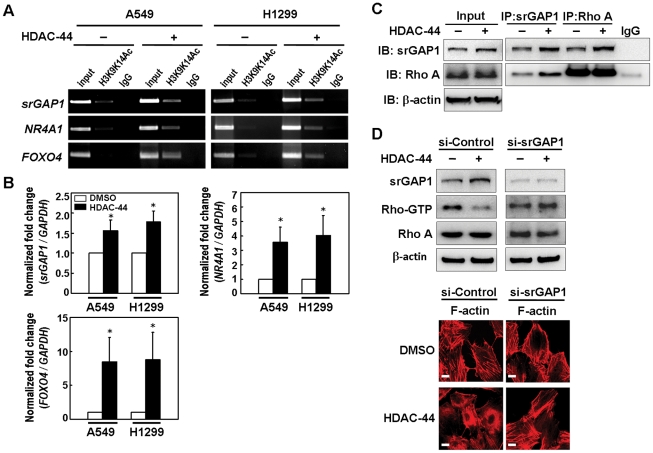
OSU-HDAC-44 decreased RhoA activity via srGAP1 induction, leading to F-actin dysregulation. (**A**) Chromatin-immunoprecipitation-PCR analyses confirmed that treatment with 2.5 µM OSU-HDAC-44 for 2 h induced acetylation of histone H3 (H3K9K14Ac) in the promoter region of *srGAP1*, *NR4A1* and *FOXO4* genes. (**B**) OSU-HDAC-44 increased the mRNA levels of *srGAP1*, *NR4A1* and *FOXO4* genes using real-time RT-PCR analyses. Cells were treated with 2.5 µM OSU-HDAC-44 for 24 h and total RNA was extracted for the real-time RT-PCR analyses. Data represent mean ± SEM from three independent experiments. *P<0.05. (**C**) Immunoprecipitation assay indicated that increased interaction between srGAP1 and RhoA was induced by OSU-HDAC-44. A549 cells were treated or untreated with 2.5 µM OSU-HDAC-44 for 24 h and subjected to IP-Western analyses. (**D**) si-srGAP1 abrogated the OSU-HDAC-44-induced decrease in RhoA activity (*upper*) and rescued the normal structure of F-actin after OSU-HDAC-44 treatment (*lower*). A549 cells transfected with srGAP1 siRNA were treated with 2.5 µM OSU-HDAC-44 for 24 h and subjected to RhoA activation assay and immunofluorescence analyses. Scale bars: 30 µm.

To determine the direct effects of OSU-HDAC-44 on chromatin structure and gene expression, the chromatin-immunoprecipitation (ChIP)-on-chip analysis was performed using the antibody against the loose chromatin mark, acetylated lysines 9 and 14 of histone H3 (H3K9K14Ac), after 2.5 µM OSU-HDAC-44 treatment for 2 hours in A549 and H1299 cells. Induction of histone acetylation in 33 common gene loci of A549 and H1299 were identified after OSU-HDAC-44 treatment (Table S1). Several of these 33 genes had been demonstrated to play important roles in certain signaling pathways, such as apoptosis, oxidative stress response, axon guidance and protein ubiquitination pathways (Table 1). To confirm microarray data, we validated the chromatin structure of some of the gene loci including *srGAP1*, *NR4A1* and *FOXO4* by ChIP-PCR using the antibody against H3K9K14Ac. As shown in Fig. 4A, treatment with 2.5 µM OSU-HDAC-44 for 2 hours increased the amount of *srGAP1*, *NR4A1* and *FOXO4* promoter DNA with loose chromatin structure compared to untreated cells. Concordantly, the mRNA levels of *srGAP1*, *NR4A1* and *FOXO4* were increased after OSU-HDAC-44 treatment for 24 hours (Fig. 4B).

**Table 1:** The signal pathways involved of 12 common genes from the ChIP-on-chip analysis of A549 and H1299 lung cancer cells.

Symbol*	Signaling pathway	Accession no.
BAP1	Protein ubiquitination pathway	BC001596
CAMKK1	Calcium signaling	NM_172206
CLYBL	Citrate cycle	NM_138280
DNAJB11	NRF2-mediated oxidative stress response	NM_016306
FOXO4	PTEN signaling	NM_005938
GPT2	Glutamate metabolism; Alanine and aspartate metabolism	NM_133443
HES7	Notch signaling	NM_032580
NEU1	Sphingolipid metabolism; N-glycan degradation	BC011900
NR4A1	Calcium-induced T lymphocyte apoptosis	BC016147
PSMB8	Protein ubiquitination pathway	BC001114
SH3BP4	Clathrin-mediated endocytosis	BC057396
SRGAP1	Axon guidance	NM_020762

*Full name was shown in Table S1, available online.

### OSU-HDAC-44 down-regulates F-actin dynamics via srGAP1 induction

OSU-HDAC-44 treatment induced F-actin aggregation (Fig. 2B). Previous study has indicated that srGAP1 binds to the active forms of RhoA and Cdc42 and inhibits their activities in regulating actin polymerization in neuron cells [19]. However, the biological function of srGAP1 binding to RhoA remains unclear in other cells. Using immunoprecipitation (IP)-Western, we showed that OSU-HDAC-44 increased the interaction between srGAP1 and RhoA in A549 lung cancer cells (Fig. 4C). Interestingly, knockdown of srGAP1 not only abolished the OSU-HDAC-44-mediated decrease in RhoA-GTP level (Fig. 4D, upper), but also restored the dynamics of F-actin after OSU-HDAC-44 treatment (Fig. 4D, lower). These results indicated that OSU-HDAC-44 down-regulated RhoA activity partly via srGAP1 induction, leading to destruction of normal F-actin fibers.

### OSU-HDAC-44 inhibits lung tumor xenograft growth *in vivo*

**Figure 5:**
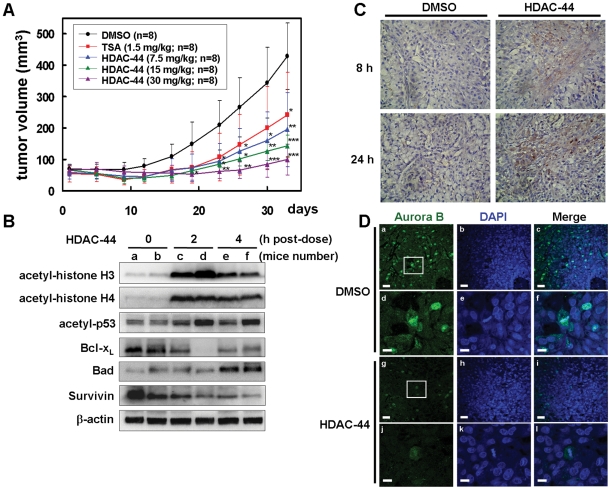
OSU-HDAC-44 effectively induced apoptosis and inhibited A549 xenograft growth. (**A**) Mice bearing the established A549 tumors (∼50 mm^3^) were injected intraperitoneally with 7.5, 15 or 30 mg/kg of OSU-HDAC-44 or 1.5 mg/kg of TSA 3 days/week for three weeks. Eight mice per group were used in the xenograft experiment. The tumor volumes of mice were measured. Points, mean; error bars, 95% confidence intervals. *P* values were for comparisons with vehicle control (*P<.05; **P<.01; ***P<.001). (**B–D**) Mice bearing established (about 100∼200 mm^3^) A549 tumors were injected intraperitoneally with a single dose of OSU-HDAC-44 at 60 mg/kg. After treatment for the indicated time, tumors were harvested and subjected to Western blot or immunohistochemistry analyses. (**B**) Tumors from two representative mice of each time point (a–f) were harvested and subjected to Western blot analyses using the indicated antibodies. (**C**) Immunohistochemistry analyses were performed using antibody against cleaved-form of caspase 3 (brownish color). Original magnification ×200. (**D**) Fluorescence immunohistochemistry analyses were performed using antibody against Aurora B and DAPI (DNA). The images (d–f and j–l, scale bars: 10 µm) were magnified from framed ones (a–c and g–I, scale bars: 30 µm).

**Figure 6:**
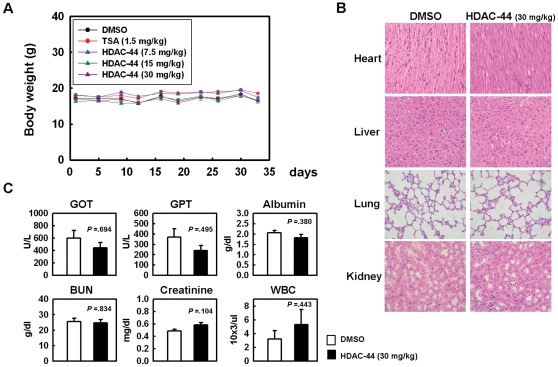
The body weight, H&E staining of major organs, and hematological biochemistry examinations of tested animals. (**A**) OSU-HDAC-44 treatments did not cause significant body weight loss of tested animals. (**B**) H&E staining of paraffin-embedded, 5 µm thick sections of the heart, liver, lung and kidney from OSU-HDAC-44-treated and untreated mice with A549 xenografts. There were no apparent histopathologic differences between these tissues sections (original magnification ×200). (**C**) Hematological biochemistry tests including GOT, GPT, albumin, BUN, creatinine, and WBC were examined and the results showed no significant differences between DMSO and OSU-HDAC-44 treatment.

To further evaluate the antitumor activity of OSU-HDAC-44, Bulb/c null mice bearing A549 lung tumor xenograft were injected intraperitoneally with 7.5–30 mg/kg of OSU-HDAC-44, 3 days/week for three weeks. TSA of 0.5 mg/kg, which has been demonstrated to exhibit anti-tumor growth effects in xenograft of breast and bladder cancer cells [20], [21], was used as a positive control drug. As shown in Fig. 5A, treatment with 7.5, 15 and 30 mg/kg OSU-HDAC-44 significantly inhibited tumor growth by 62%, 78% and 90%, respectively, on day 33 post-treatment compared with vehicle control. Treatment with OSU-HDAC-44 did not adversely affect body weight and caused no detectable toxicity as examined by hematoxylin and eosin staining of major organs (Fig. 6A, B). Hematological biochemistry examinations were in the normal ranges for OSU-HDAC-44 treated animals (Fig. 6C).

### OSU-HDAC-44 induces protein acetylation, apoptosis and cytokinesis inhibition *in vivo*

To confirm that OSU-HDAC-44 suppressed xenograft tumor growth via targeting the HDACs and inducing apoptosis *in vivo*, mice bearing established A549 tumors were treated with a single dose of OSU-HDAC-44. After treatment for indicated time, tumors were dissected and subjected to Western blot, immunohistochemistry or fluorescence immunohistochemistry analysis (Fig. 5B–D). Acetylation of histone H3, histone H4 and p53 were profoundly increased after 2 hours treatment. The protein levels of Bcl-x_L_ and survivin started to decrease after 2 hours treatment, while the level of Bad protein was increased after 4 hours treatment (Fig. 5B). Activated caspase-3 was also detected in both the cytosol and nucleus after 8 hours treatment and was further enhanced after 24 hours treatment (Fig. 5C). Furthermore, OSU-HDAC-44 decreased Aurora B levels and interrupted its association with metaphase chromosome in comparison with DMSO control cells (Fig. 5D). These results demonstrated that OSU-HDAC-44 could induce apoptosis and down-regulate mitotic and cytokinesis regulators, Aurora B and survivin, *in vivo*. In addition, increase of HDAC inhibition biomarkers such as acetylation of histone H3, histone H4 and p53 was evident in tumors of treated mice.

## Discussion

Since HDACs are promising targets for cancer therapy, a number of HDAC inhibitors are in clinical trials as single therapy and/or in combination with other anticancer drugs [9]. However, effective HDAC inhibitors for treatment of solid tumors remain to be developed. In this study, we provide compelling evidence from cell and animal studies that OSU-HDAC-44, a phenylbutyrate-based compound, is a potential HDAC inhibitor for NSCLC treatment. OSU-HDAC-44 targeted numerous members within three classes of HDACs *in vitro* and efficiently stimulated protein acetylation in cell and animal models (Fig. 3 and 5). OSU-HDAC-44 repressed cell viability and induced apoptosis in various NSCLC cell lines with 3–4 times greater potency than SAHA (Fig. 1C and 2A). In addition, submicromolar concentration of OSU-HDAC-44 exhibited prominently synergistic effects in combination with cisplatin on suppressing proliferation of NSCLC cell lines (Fig. 1D). The xenograft experiments further confirmed that OSU-HDAC-44 induced cell apoptosis and thereby inhibited tumor growth *in vivo* (Fig. 5) without adversely affected body weight, major organs and hematological parameters (Fig. 6). Collectively, these results suggested that OSU-HDAC-44 is a promising candidate HDAC inhibitor for NSCLC treatment.

It has been shown that several kinases and regulatory proteins, such as Aurora B, suvivin as well as small GTPase RhoA are required to complete cytokinesis [22]. Inhibition of Aurora B or depletion of survivin can prevent the late steps of cytokinesis, leading to formation of multi-nucleated cells [15], [16]. In the current study, we provided evidence that OSU-HDAC-44 induced proteolysis of Aurora B and survivin both *in vitro* and *in vivo* (Fig. 2C and Fig. 5B, D), which was associated with OSU-HDAC-44-mediated cytokinesis inhibition, resulting in the accumulation of bi-nucleated cells (Fig. 2B and Fig. S1A–B). In addition, combination of a pre-metaphase inducer nocodazole and OSU-HDAC-44 resulted in decrease of Aurora B and survivin protein levels upon 24 h post-treatment (Fig. S1E). These data suggested that OSU-HDAC-44-mediated cytokinesis defect was due to abnormal degradation of Aurora B and survivin in mitotic phase. It has been reported that overexpression of Aurora B correlates with survivin expression in the nucleus, lymph node invasion, and poor prognosis in NSCLC patients [23]. Thus, the clinical efficacy of OSU-HDAC-44 in relation to down-regulated Aurora B and surivin in treatment of NSCLC patients is worthy of further investigation.

In this study, we performed a ChIP-on-chip analysis to investigate the genome-wide target genes induced by OSU-HDAC-44-mediated hyperacetylation of chromatin after 2 hours exposure, and found that histone acetylation were stimulated in 33 common genes in the cell lines examined, including eight tumor suppressor genes (TSGs) or TSG-like genes (Table S1). Several genes play essential roles in apoptosis, oxidative stress response, axon guidance and protein ubiquitination pathways (Table 1). The *srGAP1* gene, which encodes a GTPase activating protein known to regulate axon guidance [19], was confirmed to be in the open chromatin structure and increased in expression level (Fig. 4A, B). Interestingly, we found that OSU-HDAC-44 decreased the activity of a small GTPase RhoA via induction of srGAP1 and contributed to dysregulation of F-actin dynamics (Fig. 4C, D). These results indicated that OSU-HDAC-44 may interrupt mitosis and cytokinesis resulting from alteration of several additional pathways, such as srGAP1/RhoA/F-actin control. Moreover, two apoptosis-related genes, *NR4A1/Nur77* and *FOXO4*, were validated from the ChIP-on-chip data and their mRNA expressions were indeed increased by OSU-HDAC-44 (Fig. 4A, B). NR4A1/Nur77 and FOXO4 have been shown to trigger intrinsic apoptosis through induction of mitochondrial cytochrome c release and down-regulation of Bcl-x_L_ expression, respectively [24]–[26]. Such NR4A1/Nur77-mediated apoptosis has been demonstrated to be induced by an HDAC inhibitor, LBH589, in CTCL cells [27]. Our results from cell and animal models showed that the OSU-HDAC-44-induced cell death was possibly through the intrinsic apoptotic pathway (Fig. 2D and 5B). Therefore, the transcriptional up-regulation of *NR4A1/Nur77* and *FOXO4* may contribute to OSU-HDAC-44-mediated intrinsic apoptosis.

Similar to our finding of selective chromatin change of a fraction of gene loci in ChIP-on-chip, recent studies using cDNA microarrays indicate that several HDAC inhibitors such as TSA, SAHA, MS-275 and depsipeptide alter only 7–20% gene expressions in various cancer cell lines [28]–[30]. Specific recruitment of corepressor complexes containing HDACs by transcription factors and/or transcription regulators is believed to play an essential role in transcriptional repression [31]–[33], however, the selective action of HDAC inhibitors on specific genes remains unclear. Thus, it is worthy to investigate whether there may be common and critical transcription-regulatory complexes containing HDACs that determine the acetylation levels of chromatin of these genes validated from ChIP-on-chip data.

**Figure 7:**
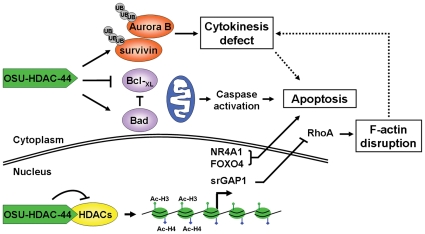
The antitumor activity of OSU-HDAC-44 via cytokinese defect, F-actin disruption, apoptosis induction, and gene acetylation. OSU-HDAC-44 is a novel pan-HDAC inhibitor that exhibits a broad spectrum of antitumor activities in NSCLC cell and xenograft models, which involves histone acetylation-dependent activation of gene transcription in nucleus. For example, re-expression of NR4A1 and FOXO4 along with caspase activation induces intrinsic apoptosis. In addition, RhoA/F-actin motility control is inhibited by srGAP1 resulting from activation by OSU-HDAC-44. OSU-HDAC-44 also induces post-translational down-regulation of mitotic regulators, Aurora B and survivin leading to cytokinese defect and apoptosis.

In conclusion, our findings shows that OSU-HDAC-44 is a novel pan-HDAC inhibitor that exhibits a broad spectrum of antitumor activities in NSCLC cell and xenograft models, which involves not only histone acetylation-dependent activation of gene transcription, but also activation of intrinsic apoptotic pathways and post-translational down-regulation of mitotic regulators, Aurora B and survivin. In addition, RhoA/F-actin motility control was inhibited by srGAP1 and several apoptosis induction proteins were activated by OSU-HDAC-44 (Fig. 7). Collectively, our data provide compelling evidence that OSU-HDAC-44 is an HDAC targeted inhibitor and has the potential to be tested for NSCLC treatment and combination chemotherapy.

## Materials and Methods

### Cell lines and culture conditions

Human normal lung cell line IMR90 and human NSCLC cell lines A549 and H1299 were obtained from the American Type Culture Collection (ATCC, Manassas, VA), and the human NSCLC cell line CL1-1 was kindly provided by Dr. P-C Yang (Department of Internal Medicine, National Taiwan University Hospital, Taipei, Taiwan) [34]. All cell lines were cultured in Dulbecco's Modified Eagle's Medium (GIBCO, Grand Island, NY) containing 10% fetal bovine serum (FBS) (BIOCHROM AG, Leonorenstr, Berlin, Germany) and 1% penicillin-streptomycin (GIBCO) and incubated at 37°C in 5% CO_2_ atmosphere.

### Preparation of OSU-HDAC-44

Isobutyric acid (1.4 mL) was added dropwise to a mixture of diisopropylamine (2.2 mL, 0.015 mol) and 54% sodium hydride in mineral oil (0.74 g, 0.0165 mol) in THF (40 mL), and refluxed for 15 min. When the solution was cooled to 0°C, a standard solution of *n*-butyllithium in heptane (1.45 mmol/mL; 9.4 mL) was added. After 20 min at 0°C, the mixture was heated to 30–35°C for 30 min and then cooled to 0°C when (2-bromoethyl)-benzene (2.8 mL, 15 mmol) was added to the reaction mixture over 20 min. The ice-bath was retained for 30 min, the mixture was then heated to 30–35°C for 1 h, and then 40 mL of water was added to the reaction mixture at a temperature below 15°C. The aqueous layer was separated, and the organic layer was washed with a mixture of 20 mL of water and 30 mL of ethyl ether. Aqueous layers were combined, back extracted with 20 mL of ethyl ether then acidified with 1N hydrochloric acid and the product was extracted with 30 mL of ethyl ether twice. The combined organic layer was washed with 20 mL of saturated brine, dried with Na_2_SO_4_, and concentrated under vacuum. Hexane was added to the resulting colorless oil to yield 1.1 g of white solid 2, 2,-Dimethyl-4-phenylbutyric acid compound. Oxalyl chloride (2 mmol) was added to the cooled solution of 2, 2,-Dimethyl-4-phenylbutyric acid (1 mmol) in dichloromethane (5 mL), and the reaction mixture was then brought to room temperature and stirred for 4 h. After the completion of the reaction, solvent was removed under vacuum. The residue was dissolved in dichloromethane (10 mL) and cooled to 0°C. Paraamino benzoic acid was then added to the reaction mixture followed by addition of triethyl amine. Resultant mixture was brought to room temperature and stirred overnight. Reaction mixture was then concentrated and purified by column chromatography to give 4-(2, 2-dimethyl-4-phenylbutanamido) benzoic acid compound. The cooled 4-(2, 2-dimethyl-4-phenylbutanamido) benzoic acid compound (1 mmol) in DMF (1 mL) was added triethyl amine (1.2 mmol) followed by PyBOP (1.2 mmol). Resultant mixture was stirred at room temperature for 4 h. After complete consumption of starting material as evidenced by TLC, reaction mixture was cooled to 0°C and hydroxylamine hydrochloride (1.2 mmol) was added to the reaction mixture followed by addition of triethyl amine (1.5 mmol). Resultant mixture was stirred at room temperature overnight and then quenched with water. Solid was filtered and purified by column chromatography to give OSU-HDAC-44.

### Analysis of cell viability

Cells were seeded in 6-well plates and treated with various concentrations of OSU-HDAC-44 or SAHA for 48 h, then stained with Trypan Blue solution (0.4%) (Sigma-Aldrich, St. Louis, MO) to measure their effects on cell proliferation. For its synergistic effect in combination with cisplatin (Bristol-Myers Squibb Caribbean Company, New York, NY), CL1-1 and A549 cells were exposed to cisplatin alone for 4 h, OSU-HDAC-44 alone for 48 h, or pretreated with OSU-HDAC-44 for 48 h before cisplatin treatment for 4 h, and then drug-containing media were replaced by drug-free media. Treated cells were incubated for additional 48 h and cell viability was assessed by Trypan Blue exclusion assay. The concentrations of drugs were described as follows: CL1-1 cells were treated with 4.4 µM cisplatin and/or 0.3 µM OSU-HDAC-44; A549 cells were treated with 1.6 µM cisplatin and/or 0.2 µM OSU-HDAC-44. For elucidation of the OSU-HDAC-44-induced cell death, phosphatidylserine (PS) staining analyses were performed and described in the Supplementary Methods S1.

### Cell cycle analysis

Cell cycle distribution was determined by flow cytometry. Cells (2×10^6^) were treated with 2.5 µM OSU-HDAC-44 or 5 µM SAHA for 24 or 48 h. Cells were trypsinized and fixed with 70% ethanol for at least 2 h at −20°C. Fixed cells were stained with a solution containing 20 µg/ml propidium iodide, 200 µg/ml RNase A, and 0.1% Triton X-100 for 20 minutes in the dark. Cell cycle distribution was performed by FACScan flow cytometry (BD Biosciences, Mountain View, CA) and calculated using ModFIT LT 2.0 version software (BD Biosciences). For examination of the cellular consequences of OSU-HDAC-44-mediated accumulation of 4N cells, time-lapse microscopic analyses were performed and described in the Supplementary Methods S1.

### Caspase activity assay

Caspase activity was measured with the caspase luminescent assay kit (Promega, Madison, WI) according to the manufacturer's instructions. Briefly, cells were plated in a 96-well plates and treated with 2.5 µM OSU-HDAC-44 for 12 or 24 h, followed by incubating with various synthetic caspase substrates (Ac-DEVD-pNA, Ac-LETD-pNA, and Ac-LEHD-pNA) to measure the activity of caspases−3, −8, and −9, respectively. After incubation for 1 h, luminescence was detected on a SpectraMax® M5 microplate reader (Molecular Devices, Sunnyvale, CA).

### siRNA transfection

The *srGAP1* siRNAs were purchased from Invitrogen (Carlsbad, CA). Cells were transfected with 300 *n*M of *srGAP1* siRNA duplexes (sense, 5′- AAA CGU AUC AUC CAU AUC CUG CAC C -3′ and antisense: 5′- GGU GCA GGA UAU GGA UGA UAC GUU U -3′) using Lipofectamine 2000 (Invitrogen) according to the manufacturer's protocols. After transfection for 48 h, the cells were subjected to OSU-HDAC-44 treatment.

### Western blot analysis

Cells were lysed on ice using RIPA buffer (0.05 M Tris-HCl, pH 7.4, 0.15 M NaCl, 0.25% deoxycholic acid, 1% NP-40, 1 mM EDTA, 0.5 mM DTT, 1 mM phenylmethylsulfonyl fluoride, 5 µg/ml leupeptin, 10 µg/ml aprotinin). The lysate was centrifuged at 13000 r.p.m for 15 minutes at 4°C. Protein extracts were solubilized in SDS gel loading buffer (60 mM Tris-base, 2% SDS, 10% glycerol, and 5% β-mercaptoethanol). Samples containing equal amounts of protein (50 µg) were separated on an 8% SDS-PAGE and electroblotted onto Immobilon-P membranes (Millipore Co., Bedford, MA) in transfer buffer. Immunoblotting was performed for various proteins, using the conditions described in the Table S2, available online. Antibody reaction was visualized using Western blot chemiluminescence reagent (Millipore).

### Immunoprecipitation assay

Catch and Release Reversible Immuonprecipitation System kit (Upstate, Temecula, CA) was used for protein-protein interaction analysis. One mg cell protein lysates were incubated with the appropriate antibodies, including anti-srGAP1, anti-RhoA, anti-Aurora B, anti-survivin or normal mouse-IgG, and 10 µl affinity ligand, and immunoprecitation was then performed according to the manufacturer's protocol. After incubation at 4°C overnight, immune complexes were washed with wash buffer for three times. Proteins were eluted and then blotted with appropriate antibodies using the conditions described in the Table S2.

### RhoA activation assay

The RhoA activation assay was performed by using active Rho pull-down and detection Kit (Pierce, Rockford, IL). Briefly, a glutathione S-transferase (GST) fusion protein containing the Rho binding domain (RBD) from Rhotekin was used. One mg protein lysates were incubated with 400 µg of purified GST-Rhotekin-RBD immobilized on agarose-glutathione beads for 1 hour at 4°C with constant agitation. The beads were washed three times with 1X Lysis/Wash buffer and bound proteins were eluted and subjected to Western blot analysis using RhoA antibody described in the Table S2.

### Molecular docking analysis

In order to show the interaction between OSU-HDAC-44 and HDAC, molecular docking assay was conducted. The reference compound, SAHA, was included. We calculated the structure of OSU-HDAC-44 and SAHA and predicted the docking mode on catalytic domain of HDAC8 using the docking program GOLD 4.0.1 to confirm the accuracy of this prediction program. The three dimensional structure of OSU-HDAC-44, the binding affinity of OSU-HDAC-44 to HDAC8, and the angles of OSU-HDAC-44 and HDAC8 were calculated by this prediction program, with consideration of molecular interaction, such as hydrogen bound and van der Waals force.

### HDAC inhibition Assay

Different HDAC isotypes were immunoprecipitated from nuclear extract using specific anti-HDAC-1, −4, −6, −8, and −11 antibodies. The HDAC activity assay was performed using a HDAC fluorescent activity assay kit (BIOMOL Inc, Plymouth Meeting, PA) according to the manufacturer's instructions. Briefly, the specific HDAC isotypes were added to the diluted OSU-HDAC-44 (1 µM) and SAHA (1 µM), and then the substrate was added. Samples were incubated for 10 min at 25°C, followed by adding developer to stop the reaction. After incubation for 10 min, luminescence was recorded on a SpectraMax® M5 microplate reader (Molecular Devices, Sunnyvale, CA).

### Target promoter chromatin immunoprecipitation (ChIP)-PCR Assay

Treated and untreated lung cancer cells were cross-linked with 1% formaldehyde for 15 min at 37°C. Chromatin was immunoprecipitated with anti-acetylated lysine 9 and lysine 14 of histone H3 (H3K9K14Ac) using the ChIP assay kit (Upstate) according to the manufacturer's instructions and the conditions were described in the Table S2. PCR analysis for protein-DNA complex was performed using the following primer pairs: *srGAP1* promoter, forward, 5′- TTT CCA TAC CAT CGC TTT CC -3′, and reverse, 5′- AAA CCC CTT CCT GAC CTG AG -3′; *NR4A1* promoter, forward, 5′- GAC CTT CAG CAA GTG CCA TT -3′, and reverse, 5′- GCC CCT GAG ACG TCA GTT AG -3′; *FOXO4* promoter, forward, 5′- GCA GAG ATG GGT TTC ACC AT -3′, and reverse, 5′- TCT CCA ACG GCT TCA CTT CT -3′.

### Chromatin structure profiling assay: ChIP-on-chip assay

The A549 and H1299 cells (4×10^6^) were treated with DMSO or OSU-HDAC-44 for 2 h, and then immunoprecipitated using antibody to H3K9K14Ac as the conditions described in the Table S2. DNA was amplified and labeled by ligation-mediated PCR with Cy5 and Cy3 fluorescent dyes, respectively. Both pools of labeled DNA were hybridized to the NimbleGen human 385k RefSeq Promoter array (Roche NimbleGen Inc., Madison, WI). Images of fluorescence intensities were generated by scanning array using GenePix 4000B scanner (Axon Instruments, Union City, CA), and then data were extracted and ChIP signals were normalized using NimbleGen SignalMap software. The ChIP-on-chip data discussed in this publication have been deposited in NCBI's Gene Expression Omnibus and are accessible through GEO Series accession number GSE20304 (http://www.ncbi.nlm.nih.gov/geo/query/acc.cgi?acc=GSE20304).

### Real-time RT-PCR assay

Expression levels of *srGAP1*, *NR4A1* and *FOXO4* mRNA were assayed by real-time RT-PCR analysis using the *GAPDH* gene as an internal control. The primers used in real-time RT-PCR are as follows: *srGAP1*, forward, 5′- GGA TGG CCC TGT TTA TGA GA -3′ and reverse: 5′- CCG CCC AAC ATA GTC AAA CT -3′; *NR4A1*, forward, 5′- GGC ATG GTG AAG GAA GTT GT -3′ and reverse: 5′- GCC TGG CTT AGA CCT GTA CG -3′; *FOXO4*, forward, 5′- CTT TGA GCC AGA TCC CTG AG -3′ and reverse: 5′- TTC CAA CAG CAT TGC TCA TC -3′; *GAPDH*, forward: 5′- AAT CCC ATC ACC ATC TTC CA -3′ and reverse: 5′- CCT GCT TCA CCA CCT TCT TG -3′. Relative quantitation using the comparative Ct method with the data from ABI PRISM 7000 (version 1.1 software) was performed according to the manufacturer's protocol. Analysis of *p21* gene expression and its primer sequence are described in the Supplementary Methods S1.

### Immunofluorescence and confocal microscopic analysis

To stain for DNA and F-actin, the fixed cells were stained with DAPI and Phalloidin, respectively, for 1 hour and then the images were recorded by an OLYMPUS FV1000 confocal microscope (Olympus America Inc., Melville, NY). For examination of the degradation of Aurora B and survivin at mitotic phase by OSU-HDAC-44, nocodazole was used to synchronize cell and then cells were subjected to immunofluorescence and confocal microscopic analysis as described in the Supplementary Methods S1.

### Xenograft studies

Athymic nu/nu female mice (BALB/c), 4–5 weeks of age, were obtained from the National Laboratory Animal Center (Republic of China, Taiwan) after being approved by Institutional Animal Care and Use Committee (IACUC), National Cheng Kung University (IACUC Approval No. 99131) and maintained in pathogen free conditions. Eight mice per group were used in the xenograft studies. The animals were implanted subcutaneously with 5×10^6^ A549 cells in 0.1 ml Hanks' balanced salt solution (HBSS) in one flank per mouse. The tumor size was measured according to the formula: (Length×Width^2^)/2. When tumors had attained a mass of ∼50 mm^3^, animals were treated intraperitoneally with OSU-HDAC-44 (7.5 mg/kg, 15 mg/kg or 30 mg/kg), TSA (1.5 mg/kg) or DMSO as control on days 1, 3, and 5 for three weeks. Prior to being sacrificed, the animals were anesthetized and blood samples were collected by intracardiac puncture for the hematological biochemistry tests. Tumor samples and mice organ tissues were resected, fixed and embedded in paraffin for histologic examination. To examine the biological effects of HDAC inhibition in tumors, mice bearing established (about 100∼200 mm^3^) A549 tumors were treated intraperitoneally with a single dose of OSU-HDAC-44 at 60 mg/kg. After treatment for indicated time, tumors were harvested and subjected to Western blot or immunohistochemistry analyses.

### Immunohistochemistry (IHC) and fluorescence IHC assays

Tumor tissues from mice exposed to OSU-HDAC-44 were analyzed using IHC assay to detect the expression levels of cleaved caspase-3 protein and were also used for immunofluorescence and confocal microscopic analysis of Aurora B where DAPI was used to stain the DNA. The conditions were as described in the Table S2.

### Statistical analysis

The SPSS program (SPSS Inc. Headquarters Chicago, Illinois) was used for all statistical analysis. Statistical analysis was performed using Student's *t*-test. Data shown were representatives of at least three independent experiments. Data represent mean ± SEM. P<0.05 was considered to be statistically significant.

## Supporting Information

Figure S1 Effect of OSU-HDAC-44 on cell cycle progression and cell cycle-regulatory proteins. (A) The cells were treated with DMSO or 2.5 µM OSU-HDAC-44 for 12 h and then subjected to time-lapse microscopy analysis. Representative images are shown for the indicated times. Arrows pointed to the dividing cells. (B) Cells were treated with (+) or without (-) 2.5 µM OSU-HDAC-44 for 24 h or 48 h. The mean percentage of bi-nucleated cells was calculated by counting over 250 cells per experiment and plotted in the histogram. Data represent mean ± SEM from three independent experiments. *, P<0.05; **, P<0.01. (C) Cells were treated with or without 2.5 µM OSU-HDAC-44 for the indicated times and blotted for the indicated proteins. (D) Cells were treated with 200 ng/ml nocodazole and/or 2.5 µM OSU-HDAC-44 for 24 h, and then subjected to immunofluorescence analyses using antibodies against Aurora B (red), survivin (green), and DAPI (blue). Scale bars: 20 µm. (E) Cells were treated with 200 ng/ml nocodazole and/or 2.5 µM OSU-HDAC-44 for indicated times and blotted for the indicated proteins.
(1.82 MB TIF)

Figure S2 OSU-HDAC-44 induced translocation of phosphatidylserine (PS) to the outer leaflet of the plasma membrane. A549 and H1299 cells were treated with 2.5 µM OSU-HDAC-44 24 h, and then subjected to immunofluorescence analyses using antibody against phosphatidylserine. Scale bars: 1.0 mm.
(0.99 MB TIF)

Figure S3 Effect of OSU-HDAC-44 on the biomarkers associated with HDAC inhibition. H1299 cells were treated with or without 2.5 µM OSU-HDAC-44 or 5 µM SAHA for the indicated times. Lysates were prepared and blotted for the indicated antibodies by Western blot analyses. The immunoblots shown are representatives of three independent experiments. Ac-H3, acetylated histone H3; Ac-H4, acetylated histone H4.
(0.55 MB TIF)

Figure S4 OSU-HDAC-44 increased p21 mRNA and protein levels in a p53-independent manner. (A) A549 (p53 wild-type) and H1299 (p53 null) cells were treated with or without 2.5 µM OSU-HDAC-44 for the indicated times and total RNA was extracted for the quantitative RT-PCR analyses using the specific primers for p21 and GAPDH. Data represent mean ± SEM from three independent experiments. ***P<0.001. (B) Cells were treated with or without 2.5 µM OSU-HDAC-44 for the indicated times. Immunoblot analyses were performed using the indicated antibodies. The immunoblots shown are representatives of three independent experiments.
(0.45 MB TIF)

Methods S1 Supplementary Methods
(0.03 MB DOC)

Table S1 Inductions of histone acetylation in 33 common genes of A549 and H1299 lung cancer cells by OSU-HDAC-44.
(0.07 MB DOC)

Table S2 The antibodies and their reaction conditions used in the present study.
(0.11 MB DOC)
