# Supplementary material for: A Novel Histone Deacetylase Inhibitor Exhibits Antitumor Activity via Apoptosis Induction, F-Actin Disruption and Gene Acetylation in Lung Cancer
Source: PLoS One. 2010 Sep 14;5(9):e12417. doi: 10.1371/journal.pone.0012417 (PMC2939045; doi:10.1371/journal.pone.0012417)
Supplement: Methods S1 — Supplementary Methods (0.03 MB DOC) [file pone.0012417.s005.doc]

**Supplemental Information**

**A Novel Histone Deacetylase Inhibitor Exhibits Antitumor Activity via Apoptosis Induction, F-actin Disruption and Gene Acetylation in Lung Cancer**

**Yen-An Tang, Wei-Ling Wen, Jer-Wei Chang, Tzi-Tang Wei, Yi-Hung Carol Tan, Santosh Salunke, Chien-Tien Chen, Ching-Shih Chen, and Yi-Ching Wang***

**Supplementary Methods**

**Time-lapse analysis.** Live cell imaging of dividing cells was performed on a Leica AS MDW microscope (Leica, Deerfield, IL) equipped with a sealed chamber at 37^o^C and 5% CO_2_ atmosphere. Images were captured at 5 min intervals with the Leica AS MDW software.

**Nocodazole treatment, Immunofluorescence and Western blot analyses.** A549 cells were seeded onto glass coverslips and treated with 200 *n*g/ml nocodazole and/or 2.5 μM OSU-HDAC-44 for 24 h, and then cells were subjected to immunofluorescence and confocal microscopic analysis of Aurora B and survivin where DAPI was used to stain the DNA. For Western blot analyses, A549 and H1299 cells were treated with 200 *n*g/ml nocodazole and/or 2.5 μM OSU-HDAC-44 for indicated times and blotted for the indicated proteins. The conditions used were as described in the **Table S2**.

**Early apoptosis detection/phosphatidylserine (PS) staining.** Cells were seeded at a density of 1×10^5^ onto glass coverslips. Cells were treated with DMSO or 2.5 μM OSU-HDAC-44 for 24 h and incubated with the appropriate antibody following the conditions described in **Table S2.** The cells were observed with an Olympus BX50 fluorescence microscope.

**Quantitative RT–PCR assay for *p21* gene.** Expression levels of *p21* mRNA were assayed in a quantitative RT-PCR analysis using the *GAPDH* gene as an internal control. Total RNA was extracted from A549 and H1299 cells and equal amounts of RNA were reverse transcribed into cDNA. The primers used in quantitative RT-PCR are as follows: *p21*, forward, 5’- TCA CCG AGA CAC CAC TGG AG -3’ and reverse: 5’- TGG AGT GGT AGA AAT CTG TC -3’; *GAPDH*, forward: 5’- AAT CCC ATC ACC ATC TTC CA -3’ and reverse: 5’- CCT GCT TCA CCA CCT TCT TG -3’. Relative quantitation using the comparative Ct method with the data from ABI PRISM 7000 (version 1.1 software) was performed according to the manufacturer's protocol.
