# Supplementary material for: A Novel Histone Deacetylase Inhibitor Exhibits Antitumor Activity via Apoptosis Induction, F-Actin Disruption and Gene Acetylation in Lung Cancer
Source: PLoS One. 2010 Sep 14;5(9):e12417. doi: 10.1371/journal.pone.0012417 (PMC2939045; doi:10.1371/journal.pone.0012417)
Supplement: Table S1 — Inductions of histone acetylation in 33 common genes of A549 and H1299 lung cancer cells by OSU-HDAC-44. (0.07 MB DOC) [file pone.0012417.s006.doc]

| **Table S1. Inductions of histone acetylation in 33 common genes of A549 and H1299 lung cancer cells by OSU-HDAC-44** | | | |
| --- | --- | --- | --- |
| **Symbol** | **Gene name** | **Molecular function/ Biological process** | **Accession no.** |
| ANKRD16 | ankyrin repeat domain 16 | ND | NM_001009941 |
| BAP1* | BRCA1 associated protein-1 (ubiquitin carboxy-terminal hydrolase) | Negative regulation of cell proliferation | BC001596 |
| C1orf126 | chromosome 1 open reading frame 126 | ND | NM_182534 |
| C1orf89 | chromosome 1 open reading frame 89 | Small GTPase mediated signal transduction | BC008702 |
| CAMKK1 | calcium/calmodulin-dependent protein kinase kinase 1, alpha | Calmodulin-dependent protein kinase activity | NM_172206 |
| CLYBL | citrate lyase beta like | Cellular aromatic compound metabolic process | NM_138280 |
| DNAJB11 | DnaJ (Hsp40) homolog, subfamily B, member 11 | Heat shock protein binding | NM_016306 |
| FAM122A | family with sequence similarity 122A | ND | NM_138333 |
| FAM81A | family with sequence similarity 81, member A | ND | NM_152450 |
| FBXO24 | F-box protein 24 | Ubiquitin-protein ligase activity | NM_033506 |
| FLJ35220 | hypothetical protein FLJ35220 | Response to DNA damage stimulus | NM_173627 |
| FOXO4* | forkhead box O4 | Negative regulation of cell proliferation and angiogenesis | NM_005938 |
| GOLGA5 | golgi autoantigen, golgin subfamily a, 5 | Golgi vesicle transport | NM_005113 |
| GPT2 | glutamic pyruvate transaminase (alanine aminotransferase) 2 | L-alanine:2-oxoglutarate aminotransferase activity | NM_133443 |
| HES7 | hairy and enhancer of split 7 (Drosophila) | Notch signaling pathway | NM_032580 |
| KIAA1539 | KIAA1539 | ND | AK021622 |
| KLHDC3 | kelch domain containing 3 | Reciprocal meiotic recombination | BC001793 |
| LRCH4 | leucine-rich repeats and calponin homology (CH) domain containing 4 | Regative regulation of transcription | BC018529 |
| LRIG2 | leucine-rich repeats and immunoglobulin-like domains 2 | Protein binding | NM_014813 |
| MEA1 | male-enhanced antigen 1 | Cell differentiation | NM_014623 |
| MZF1* | myeloid zinc finger 1 | Transcription factor activity | NM_003422 |
| NEU1* | sialidase 1 (lysosomal sialidase) | Exo-alpha-sialidase activity | BC011900 |
| NR4A1* | nuclear receptor subfamily 4, group A, member 1 | Induction of apoptosis | BC016147 |
| P2RX6* | purinergic receptor P2X, ligand-gated ion channel, 6 | ATP-gated cation channel activity | BC033488 |
| PAPOLA | poly(A) polymerase alpha | Nucleotidyltransferase activity | BC036014 |
| PSMB8 | proteasome (prosome, macropain) subunit, beta type, 8 | Proteolysis involved in cellular protein catabolic process | BC001114 |
| SAMD12 | sterile alpha motif domain containing 12 | ND | NM_207506 |
| SFXN3 | sideroflexin 3 | Iron ion transport | CR614959 |
| SH3BP4 | SH3-domain binding protein 4 | Cell cycle | BC057396 |
| SRGAP1* | SLIT-ROBO Rho GTPase activating protein 1 | GTPase activator activity | NM_020762 |
| ZC3H12A* | zinc finger CCCH-type containing 12A | Apoptosis | NM_025079 |
| ZDHHC9 | zinc finger, DHHC-type containing 9 | Acyltransferase activity | NM_016032 |
| ZNF341 | zinc finger protein 341 | Regulation of transcription | NM_032819 |

* Tumor suppressor gene (TSG) or TSG-like gene.

ND: not determined.
