# Supplementary material for: A Novel Histone Deacetylase Inhibitor Exhibits Antitumor Activity via Apoptosis Induction, F-Actin Disruption and Gene Acetylation in Lung Cancer
Source: PLoS One. 2010 Sep 14;5(9):e12417. doi: 10.1371/journal.pone.0012417 (PMC2939045; doi:10.1371/journal.pone.0012417)
Supplement: Table S2 — The antibodies and their reaction conditions used in the present study. (0.11 MB DOC) [file pone.0012417.s007.doc]

**Table S2.** The antibodies and their reaction conditions used in the present study.

| **Target** | **K.D.** | **Raised In** | **Application** | **Dilution** | **Source** | **Catalog No.** |
| --- | --- | --- | --- | --- | --- | --- |
| Aurora B | 39 | Rabbit | Western blot | 1:2000 | Abcam | ab2254 |
|  |  |  | Immunoprecipitation | 1:500 |  |  |
|  |  |  | Immunofluorescence | 1:200 |  |  |
| Bad | 25 | Mouse | Western blot | 1:1000 | Santa Cruz | sc-8044 |
| Bcl-x_L_ | 30 | Mouse | Western blot | 1:500 | Santa Cruz | sc-8392 |
| Caspase 3  (cleaved form) | 17,19 | Rabbit | Immunohistochemistry | 1:500 | Cell Signaling | 9661 |
| Caspase 9 | 47,37 | Mouse | Western blot | 1:500 | Millipore | 05-572 |
| CDK1 | 34 | Rabbit | Western blot | 1:500 | Cell Signaling | 9112 |
| CDK2 | 35 | Rabbit | Western blot | 1:500 | Abcam | ab7954 |
| CDK4 | 34 | Rabbit | Western blot | 1:1500 | Santa Cruz | sc-601 |
| Cyclin B | 60 | Mouse | Western blot | 1:1000 | Santa Cruz | sc-245 |
| Cyclin D | 36 | Mouse | Western blot | 1:500 | Cell Signaling | 2926 |
| Cyclin E | 48 | Mouse | Western blot | 1:1000 | Cell Signaling | 41296 |
| Cytochrome c | 14 | Rabbit | Western blot | 1:800 | Epitomics | 2119-1 |
| HDAC1 | 65 | Rabbit | Western blot | 1:2000 | Millipore | 06-720 |
|  |  |  | Immunoprecipitation | 1:500 |  |  |
| HDAC4 | 140 | Rabbit | immunoprecipitation | 1:500 | Santa Cruz | sc-11418 |
| HDAC6 | 134 | Rabbit | Western blot | 1:1000 | Millipore | 07-732 |
|  |  |  | immunoprecipitation | 1:500 |  |  |
| HDAC8 | 44 | Rabbit | immunoprecipitation | 1:500 | Santa Cruz | sc-11405 |
| HDAC11 | 39 | Mouse | immunoprecipitation | 1:500 | Santa Cruz | sc-101065 |
| Histone H3 serine 10 phosphorylation | 17 | Rabbit | Western blot | 1:500 | Cell Signaling | 9701 |
| Histone H3 lysine 9, 14 acetylation | 17 | Rabbit | Western blot | 1:1000 | Millipore | 06-599 |
|  |  |  | Immunoprecipitation | 1:500 |  |  |
| Histone H4 acetylation | 17 | Rabbit | Western blot | 1:1000 | Millipore | 06-598 |
| p21 | 21 | Mouse | Western blot | 1:1000 | Santa Cruz | sc-817 |
| p53 | 53 | Mouse | Western blot | 1:2000 | Santa Cruz | sc-126 |
| p53 lysine 379 acetylation | 53 | Rabbit | Western blot | 1:500 | Cell Signaling | 2570 |
| Phalloidin/F-actin | -- | -- | Immunofluorescence | 1:50 | Invitrogen | A22287 |
| Phosphatidylserine | -- | Mouse | Immunofluorescence | 1:200 | Millipore | 05-719 |
| RhoA | 21 | Rabbit | Western blot | 1:500 | Santa Cruz | sc-179 |
|  |  |  | immunoprecipitation | 1:500 |  |  |
| srGAP1 | 130 | Mouse | Western blot | 1:1000 | Abnova | H00057522 |
|  |  |  | immunoprecipitation | 1:500 |  |  |
| Survivin | 16.5 | Mouse | Western blot | 1:500 | Santa Cruz | sc-17779 |
|  |  |  | Immunoprecipitation | 1:500 |  |  |
|  |  |  | Immunofluorescence | 1:100 |  |  |
| Ubiquitin | -- | Mouse | Western blot | 1:1000 | Santa Cruz | sc-8017 |
| Wee1 | 95 | Rabbit | Western blot | 1:500 | Cell Signaling | 4936 |
| β-actin | 42 | Mouse | Western blot | 1:5000 | Novus Biologicals | NB 600-501 |
